# Keeping it fresh

**DOI:** 10.7554/eLife.48268

**Published:** 2019-06-12

**Authors:** Jonathan L Klassen

**Affiliations:** Department of Molecular and Cell BiologyUniversity of ConnecticutStorrsUnited States

**Keywords:** antimicrobial strategy, nitric oxide, nitric oxide synthase, mould fungi, insect egg, Philanthus triangulum, Other

## Abstract

Beewolf wasp eggs release nitrogen oxides to provide protection against fungi and other microbes.

**Related research article** Strohm E, Herzner G, Ruther J, Kaltenpoth M, Engl T. 2019. Nitric oxide radicals are emitted by wasp eggs to kill mold fungi. *eLife* **8**:e43718. doi: 10.7554/eLife.43718

Beewolves, a type of wasp that preys on bees, provide for their offspring by laying eggs on paralyzed honeybees. Each egg is laid on its own honeybee in what is known as a ‘brood cell’, which is then sealed inside a waterproof coating, buried underground and left to fend for itself. When the egg hatches the beewolf larva can use the paralyzed honeybee as a source of food for up to two weeks. A challenge for the beewolf is to keep the paralyzed honeybee ‘fresh’ until it is needed: this means that they have to prevent the honeybee from being overrun by bacteria and fungi.

Previous studies have identified two possible solutions to this challenge. First, beewolves create a low humidity environment that is unfavorable for fungal growth by secreting a waterproof coating around each paralyzed honeybee (Herzner and Strohm, 2007). Second, beewolves host a specific type of bacterium, known as ‘*Candidatus* Streptomyces philanthi’, in specialized glands on their antennae (Kaltenpoth et al., 2005). Before laying each egg, a beewolf smears secretions containing this bacterium onto the roof of each brood cell. Then, six to eight days after hatching, the beewolf larva spins a cocoon around the honeybee, incorporating the bacterium from this secretion into the cocoon’s threads. The ‘*Cand*. S. philanthi’ within the cocoon then produce several antibiotics that prevent it from becoming contaminated with fungi (Kroiss et al., 2010).

However, experimentally replicating these two mechanisms did not protect the buried honeybees to the same extent as observed in nature (Strohm and Linsenmair, 2001), indicating that there may be at least one other mechanism at work. Now, in eLife, Erhard Strohm, Gudrun Herzner, Joachim Ruther, Martin Kaltenpoth and Tobias Engl (who are based at the University of Regensburg and the Max Planck Institute for Chemical Ecology) report their discovery of this missing mechanism (Figure 1; Strohm et al., 2019).

**Figure 1.**
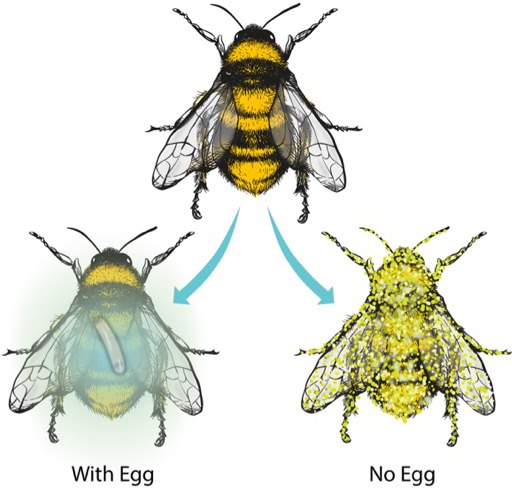
Beewolf eggs emit nitrogen oxides to protect against fungal contamination. When a beewolf wasp lays an egg on a paralyzed honeybee, the egg produces volatile nitrogen oxides (blue gas) that prevent the honeybee from being contaminated by fungi, thus allowing the beewolf larva to feed on it (Left). Without such protection, the honeybee quickly becomes overgrown by fungi (Right). Image credit: Virge Kask (CC BY 4.0).

Based on the simple observation that beewolf eggs, but not larvae, had a distinctive chlorine-like smell, Strohm et al. predicted that these eggs were producing a similarly volatile chemical. Subsequent analysis revealed that the eggs were releasing nitric oxide, which can spontaneously react with oxygen to produce the odorous gas nitrogen dioxide. In the insect world nitric oxide is widely used as a signaling molecule, usually at low concentrations (Müller, 1997), and some insects produce high levels of nitric oxide as part of an immune response to microbial infection (Rivero, 2006). At such high levels, nitrogen oxides (nitric oxide and its oxidation product nitrogen dioxide) are toxic to a wide range of organisms, and so are well suited to providing protection against the microbes found in soil.

Strohm et al. found that production of nitrogen oxides peaked 14 hours after a beewolf egg had been laid, which should allow high concentrations of these gases to be maintained in the brood cell throughout larval development. They also showed that nitrogen oxides by themselves strongly inhibited the growth of several different species of mold fungi, as did the nitrogen oxides released by the eggs. However, a combination of cocoon spinning and nitrogen oxide production was needed to reduce fungal contamination to the levels observed in the brood cells of wild beewolves.

Having clearly established its antifungal role, Strohm et al. went on to investigate how these exceptional levels of nitrogen oxides are produced. Sequencing revealed that the gene for NOS (the enzyme that synthesizes nitric oxide) in beewolves was highly similar to those found in other related species. However, differences in splicing (the process that removes the non-coding parts of genes following transcription) caused this enzyme to have a variant form of mRNA that Strohm et al. hypothesized could be responsible for the higher production of nitric oxide in beewolf eggs. If this is the case, however, it remains unclear how this splice variant carries out the other roles that NOS normally performs, such as signaling during development. In addition, it is unclear how the beewolf larvae, and their ‘*Cand.* S. philanthi’ symbionts, protect themselves from toxic nitrogen oxides. It may be that each antifungal mechanism is tuned to maximize overall antifungal activity throughout larval development while minimizing collateral damage to the developing beewolf itself.

Strohm et al. have added important new details to our understanding of how beewolves defend their prey within brood cells. It will be interesting to test if volatile compounds are involved in antifungal defense mechanisms in other systems, in addition to known mechanisms such as antibiotic production. Nitric oxide, and other compounds produced from the metabolism of nitrate, have long been used to prevent microbial contamination of human food (Cammack et al., 1999). Therefore, studies of brood-caring insects such as beewolves could potentially inspire new and more effective preservation strategies for use during human food production.
